# Developments in Cell-Penetrating Peptides as Antiviral Agents and as Vehicles for Delivery of Peptide Nucleic Acid Targeting Hepadnaviral Replication Pathway

**DOI:** 10.3390/biom8030055

**Published:** 2018-07-16

**Authors:** Bénédicte Ndeboko, Olivier Hantz, Guy Joseph Lemamy, Lucyna Cova

**Affiliations:** 1 Institut National de la Sante et Recherche Medicale (INSERM) U1052, Cancer Research Center of Lyon (CRCL), 69003 Lyon, France; olivier.hantz588@orange.fr (O.H.); lucyna.cova@gmail.com (L.C.); 2 Département de Biologie Cellulaire and Moléculaire-Génétique, Faculté de Médecine, Université des Sciences de la Santé, Libreville 241, Gabon; guylemamy@yahoo.fr

**Keywords:** cell penetrating peptides (CPPs), peptide nucleic acids (PNAs), drug delivery, virus, hepatitis B virus (HBV), duck hepatitis B virus (DHBV), antiviral therapy

## Abstract

Alternative therapeutic approaches against chronic hepatitis B virus (HBV) infection need to be urgently developed because current therapies are only virostatic. In this context, cell penetration peptides (CPPs) and their Peptide Nucleic Acids (PNAs) cargoes appear as a promising novel class of biologically active compounds. In this review we summarize different in vitro and in vivo studies, exploring the potential of CPPs as vehicles for intracellular delivery of PNAs targeting hepadnaviral replication. Thus, studies conducted in the duck HBV (DHBV) infection model showed that conjugation of (D-Arg)_8_ CPP to PNA targeting viral epsilon (ε) were able to efficiently inhibit viral replication in vivo following intravenous administration to ducklings. Unexpectedly, some CPPs, (D-Arg)_8_ and Decanoyl-(D-Arg)_8_, alone displayed potent antiviral effect, altering late stages of DHBV and HBV morphogenesis. Such antiviral effects of CPPs may affect the sequence-specificity of CPP-PNA conjugates. By contrast, PNA conjugated to (D-Lys)_4_ inhibited hepadnaviral replication without compromising sequence specificity. Interestingly, Lactose-modified CPP mediated the delivery of anti-HBV PNA to human hepatoma cells HepaRG, thus improving its antiviral activity. In light of these promising data, we believe that future studies will open new perspectives for translation of CPPs and CPP-PNA based technology to therapy of chronic hepatitis B.

## 1. Introduction

Hepatitis B virus (HBV) infection is still a major public health issue, with over 240 million virus carriers worldwide. Indeed, chronic HBV infection significantly increases the risk for the development of end stage liver diseases such as cirrhosis and hepatocellular carcinoma (HCC) [1]. Currently, there are two major types of anti-HBV therapeutic strategies, nucleos(t)ide analogues (NAs) that inhibit viral reverse transcriptase and the immune-based treatments such as interferon-α and pegylated-interferon alpha (pegylated-IFN-α). These antiviral therapies are only partially effective as they are unable to eradicate the viral persistence reservoir, an episomal covalently closed circular (ccc) DNA in the infected cells that is responsible for viral relapse after treatment cessation [2,3].

Moreover, the long-term use of these therapies increases the risk of drug resistance and of serious side effects. Therefore, alternative therapeutic approaches need to be urgently developed to eradicate chronic hepatitis B infection.

In recent years, cell penetrating peptides (CPP)s appeared of interest for non-viral delivery of different antisense macromolecules targeting HBV replication. Within different antisense agents, peptide nucleic acids (PNAs) provide a potentially promising approach to treat chronic HBV infection. Indeed, PNAs are DNA mimics containing an uncharged pseudopeptide backbone with *N*-(2-aminoethyl)-glycine units to which the nucleobases are attached via methylene carbonyl linkers [4]. These nucleic analogues are capable of sequence-specific recognition of DNA and RNA through Watson-Crick base pairing [5,6]. Furthermore, PNAs have attractive potential as antiviral agents because of their high hybridization affinity and specificity. Importantly, PNAs exhibit resistance to protease and nuclease degradation in the cells, fluids, and tissues, showing an exceptional stability in biological environments [4,5,7]. Thus, we have previously demonstrated a potent effect of PNA targeting of the hepadnaviral ε sequence, leading to sequence-specific inhibition of viral reverse transcription in a cell-free system [8,9].

However, the application of PNAs as antiviral therapeutics has been hampered by their poor cell uptake and biodistribution due to their uncharged structure. To overcome these difficulties, the conjugation of PNAs to CPPs appears as a strategy of choice to enhance their delivery into cells [9,10].

In this context, several studies documented the role of CPPs in intracellular delivery of biologically active cargos such as PNAs to the host tissues. Thus, different CPPs such as oligoarginine [11,12,13] and oligolysine [14,15] were shown to efficiently internalize PNAs through the cell membrane. These CPPs are small in size (less than 30 residues) and have been shown to considerably increase the cellular uptake and antisense activity of their cargos. However, data on antiviral activity of CPP-PNAs conjugates in the control of HBV infection were scarce and limited to the murine model.

We explored the ability of CPP-PNA conjugates to inhibit HBV replication. Because HBV, displays an extremely narrow host range, infecting only humans and chimpanzee, we have chosen the closely related duck HBV (DHBV) as a model virus. DHBV belongs to the Hepadnaviridae family, encompassing mammalian and avian viruses. Hepadnaviruses are small, enveloped DNA viruses that replicate in the liver of their hosts [16]. The Eastern woodchuck and Pekin duck, which are natural hosts of DHBV and woodchuck hepatitis B virus (WHV), respectively, played an important role in the understanding of the hepadnaviral replication pathway and in the in vivo evaluation of novel therapeutic approaches against chronic hepatitis B infection. Both DHBV and WHV share with the human HBV a similar genome organization and replication pathway via the reverse transcription of pregenomic RNA [16].

Interestingly, the DHBV model allows study of innovative anti-HBV strategies both in vivo in DHBV-carrier ducklings and in vitro in primary duck hepatocytes (PDH) or stably transfected cell lines. Thus, DHBV represents an attractive model system validated by us and others for the evaluation of novel anti-HBV strategies in preclinical studies [17,18,19,20,21,22].

Because the major problem of in vivo PNAs delivery is a poor cellular uptake, as a first step, we investigated in Pekin ducklings the uptake and biodistribution of fluorescein isothiocyante (FITC)-labeled PNA (FITC-PNA) administrated via different routes [10].

Next, we evaluated in this model the antiviral activity of different CPPs for delivery to hepatocytes of a PNA targeting hepadnaviral encapsidation signal (ε). The results generated in vitro in DHBV-infected PDH cultures and in vivo virus-carrier ducklings allowed identification of a CPP-PNA conjugate that specifically inhibited DHBV replication following low-dose administration [10].

Surprisingly, in a pilot study we observed an anti-DHBV activity of (DArg)_8_ CPP alone when used as a control in the absence of its PNA cargo [10,23]. Based on this unexpected finding, we have further investigated the antiviral activity of different CPPs and their lipid domain conjugates (CatLips). The detailed analysis of impact of these CatLips on different steps in hepadnaviral replication allowed us to identify their novel mechanism of action [23].

In addition, sugar-based CPP conjugated to a PNA targeting the HBV surface (S) gene was evaluated in this report. Our preliminary findings demonstrated that treatment of HBV-infected HepaRG cells by CPP-PNA conjugate coupled to Lactose inhibited HBV surface antigen (HBsAg) release.

This review focuses on the crucial role of cationic polymers as cargos and on the advantage of their modifications for intracellular delivery of PNAs targeting hepadnaviral RNA and envelope proteins. Thus, CPPs can mediate intracellular uptake of bioactive agents and consequently improve their biological activities. Moreover, some CPPs exhibit a potent antiviral activity on hepadnavirus replication, which can alter the specificity of viral inhibition displayed by their antisense PNA cargo. This antiviral activity can be strongly increased when these CPPs were modified by the fatty acid domains. In addition, the anti-HBV activity of CPP-PNA conjugated to another delivery system such as sugar moieties is also documented in this report.

## 2. Why Cell Penetrating Peptides Are of Particular Interest for Delivery of Bioactive Molecules

Various bioactive molecules, including PNAs, need to cross the cellular membrane to exhibit their activity. In this view, different CPPs also termed PTDs (for protein transduction domains), MTSs (for membrane translocation sequences), or CTPs (for cytoplasmic transduction peptides) [24,25] are short cationic sequences that are able to cross the cellular plasma membranes and transport bioactive molecules into a variety of cells without detectable toxicity [26,27,28,29].

To date, several cationic peptides have been described such as the transactivator of transcription (TAT) peptide from Human Immunodeficiency Virus’ (HIV’s) transactivator of transcription protein. TAT peptide represents the first cationic CPP discovered that was able to cross the lipid bilayer cell membrane and powerfully enter into the cells [30,31]. Thus, Song et al. reported that camptothecin (CPT) coupled to a TAT peptide (TAT-CPT and TAT-2CPT) could kill cancer cells by releasing the CPT after entering the cells [32]. In addition, the gold nanoparticles (AuNP) coupled to two derivatives of TAT peptide of 5 nm diameter (AuNP-CPPs) exhibited a strong fluorescent signal in Gram positive and Gram negative bacterial strains. The confocal microscopy data showed that the derivatives of the TAT peptide improve nanoparticle internalization into bacterial cells [33].

Identification of TAT was rapidly followed by the discovery of other cationic peptides displaying comparable uptake activity, including VP22, a herpes virus protein [34], and Antennapedia, a transcription factor from drosophila, termed penetratin (pAntp) [35,36].

Recently, a novel CPP, designated X-pep, found at the extreme N-terminus of the X-protein of the HBV has been identified. Moreover, the truncated form of this X-pep peptide (Met-Ala-Ala-Arg-Leu amino acids (MAARL) sequence) was able to readily penetrate into hepatoblastoma G2 (HepG2) cells. Further truncation by removal of the terminal leucine amino acid reduced the cell-penetrating activity of this peptide, indicating that the MAARL sequence is the active core of this peptide [37].

Importantly, the ability of these CPPs to translocate across the lipid bilayer of cell membranes was conferred by a short sequence rich in basic residues [38].

## 3. Antiviral Activity of Cell Penetrating Peptides Altering Hepadnaviral Replication

To date, the biological activities of some natural CPPs have been reported including antifungal, antitumoral, antibacterial, and antiviral activities [39,40,41,42]. However, studies investigating the antiviral activity of CPPs focused essentially on enveloped viruses such as HIV and HSV (herpes simplex virus), thus demonstrating their ability to interfere with viral entry [43,44,45,46]. By contrast, the ability of CPPs to inhibit HBV replication has not been investigated yet.

Because the efficiency of current anti-HBV treatments is only partial as they are unable to eliminate viral cccDNA, the template for transcription of pregenomic RNA and subgenomic RNAs, our team focused on the search for novel therapeutic strategies able to clear this viral minichromosome from infected hepatocytes. Interestingly, during investigation of CPP-PNAs conjugates as novel anti-HBV agents, we surprisingly discovered that (D-Arg)_8_ peptide alone was able to inhibit hepadnaviral replication [10,23]. This finding prompted us to initiate in vitro and in vivo studies in a DHBV infection model to better understand the antiviral action of such CPPs [10,23]. 

Thus, in vivo administration of 1 or 2 µg/g bw/day of (D-Arg)_8_ to DHBV-infected ducklings via an intravenous (i.v) route, resulted in a marked and dose-dependent decrease in viremia in treated animals (42–58%) as compared with the untreated controls, in the absence of toxicity (Table 1). To further explore the antiviral activity of this CPP, we have performed in vitro analysis in PDH cultures. Treatment of DHBV-infected PDH with (D-Arg)_8_ resulted in a decrease in viral DNA levels in both supernatants and cells, further confirming the in vivo generated data (Table 1). Collectively, these results indicate that antiviral activity of some CPPs such as (D-Arg)_8_ may affect the sequence-specificity of CPP-PNA conjugates [10].

Based on these results and using the same DHBV infection model, we have evaluated additional CPPs, including the lipid modified CPPs (CatLip) for their capacity to inhibit viral replication in vitro in stably DHBV-transfected chicken hepatoma cells (LMH-D2) and in PDHs. Thus, twelve oligoarginine-based CatLips, differing by their fatty domains and oligoarginine lengths, were analyzed for their antiviral activity. Our results showed that all evaluated CatLips decreased viral secretion in both LMH-D2 and PDH cells without measurable toxicity [23].

Remarkably, Decanoyl-(DArg)_8_ was identified as the most efficient inhibitor of DHBV release into the cell culture supernatant. This potent inhibition of DHBV release exhibited by Decanoyl-(DArg)_8_ peptide was not associated with toxicity (Table 1) [23]. Our results extend the findings of Li et al., demonstrating that the long fatty acid chain derivatives of (DArg)_8_ are efficient delivery agents for small interfering RNAs (siRNAs) and may facilitate their therapeutic application [47].

To study the specificity of inhibition displayed by Decanoyl-(DArg)_8_, different peptide doses were administrated daily to both LMH-D2 and PDH cells. Thus, our results showed that treatment of these cells for seven days by different concentrations of decanoyl-(DArg)_8_ peptide led to a strong and dose-dependent inhibition of viral secretion reaching 90% at 2 μM (Table 1), [23]. This inhibition rate was comparable to that obtained with the Lamivudine at 100 μM. Moreover, in another similar experiment we analyzed whether antiviral activity of decanoyl-(DArg)_8_ is caused only by the cationic (DArg)_8_ moiety or also by the alkyl chain (decanoic acid). Interestingly, the administration of (DArg)_8_ CPP led to a dose-dependent decrease of viral secretion reaching 60%, while decanoic acid alone was unable to significantly inhibit DHBV secretion [23].

Taken together, our data indicate that the antiviral activity of (DArg)_8_ CPP is considerably lower as compared to that displayed by decanoyl-(DArg)_8_ peptide in the same experimental conditions, indicating that the amphipathic molecules used to modify CPP sequences are able to improve their cellular uptake and consequently their biological activities. Interestingly, study of intracellular uptake of decanoyl-(DArg)_8_-Fluorescein isothiocyanate (FITC) and (DArg_8_)-FITC peptides using fluorescence microscopy, showed that at the same dose, decanoyl-(DArg)_8_-FITC peptide was internalized more powerfully into the PDH cultures than was (D-Arg_8_)-FITC. These data are in agreement with the greater antiviral activity of decanoyl-(DArg)_8_ as compared to the (DArg_8_) peptide [23].

To extend these promising results to the human virus, we analyzed the effect of decanoyl-(DArg)_8_ on HBV using the stably HBV-transfected cell line HepG2.2.15, which constitutively secretes this virus. Treatment of the HepG2.2.15 cell line by decanoyl-(DArg)_8_ led to a dose-dependent inhibition of HBV secretion by 50% at 4 μM (Table 1) [23].

Next, we asked whether decanoyl-(DArg)_8_ has a direct impact on DHBV infectivity and the susceptibility of PDH to viral infection, since the cationic antimicrobial peptides are known to induce a strong membrane disruption resulting in an antiviral effect. Our results revealed that decanoyl-(DArg)_8_ peptide neither directly inactivated DHBV nor interacted with its cellular receptor. Indeed, the pre-incubation of decanoyl-(DArg)_8_ peptide with a DHBV-positive inoculum or with PDH cells did not diminish virus infectivity and did not affect PDH susceptibility to viral infection [23]. These results suggest that the decanoyl-(DArg)_8_ peptide is not a ligand of hepadnaviral receptor [23,48,49].

Furthermore, we analyzed, in the same PDH and LMH-D2 cell systems, the effect of treatment by decanoyl-(DArg)_8_ on viral replication to establish whether the strong decrease in DHBV secretion was correlated with an eventual inhibition of DHBV replication. Our data showed that treatment of cells by decanoyl-(DArg)_8_ led to the accumulation of different viral DNA replicative forms, while this treatment did not affect viral DNA transcription and viral RNA translation. Indeed, decanoyl-(DArg)_8_ peptide treatment did not modify the intracellular cccDNA synthesis and the expression of the viral envelope and core proteins [23].

Additional studies using confocal laser microscopy scanning revealed that treatment by decanoyl-(DArg)_8_ of PDH and LMH-D2 cells resulted in the formation of large clusters of DHBV envelope proteins, without affecting the intracellular localization of viral core proteins. Moreover, the analysis of DHBV release, using iodixanol-based gradient centrifugation, indicated that decanoyl-(DArg)_8_ peptide treatment strongly decreased the secretion of both complete virions and subviral particles, while the naked DHBV capsids were still released [23].

Thus, these findings demonstrate that Decanoyl-(DArg)_8_ hampers the envelopment of hepadnaviral capsids. Interestingly, in another report, the synthetic recombinant CPP bearing nucleocapsid binding subunits (CPP-NBS) was shown to efficiently enter into the HepG2.2.15 cells and block viral nucleocapsid assembly, thereby inhibiting the HBV release that was associated with an increase in intracellular hepatitis B core antigen (HBcAg) and HBV DNA replicative intermediates by 2–3 fold [50].

Due to the capacity of CPPs to directly interact with the biologic lipid bilayer of the cells [51], our data suggest that the decanoyl-(DArg)_8_ peptide may indirectly induce severe structural changes in the morphogenesis of the DHBV envelope proteins that may obstruct their association with viral capsids and thus hamper virions secretion [23].

Collectively, our findings support the notion that decanoyl-(DArg)_8_, a cationic lipopeptide, is able to strongly interfere with the late stages of hepadnaviral morphogenesis and replication via a drastic blockage of complete virions and subviral particles release [23]. This is an interesting and novel concept as the antiviral mechanism of CPPs, described to date, was based on innate response activation or viral uptake inhibition [52]. These results, generated in preclinical studies, could be of particular interest for future development of novel anti-HBV therapeutic options.

## 4. Anti-Duck Hepatitis B Virus Effect of Peptide Nucleic Acids coupled to Cell Penetrating Peptides Conjugates

Antisense strategies appear to have promise for the control of chronic viral infections. In this view, we initially evaluated the antiviral potential of different antisense molecules such as phosphodiester oligodeoxynucleotides (ODNs), targeting the DHBV large envelope gene. Using the DHBV infection model, we demonstrated that such antisense ODNs complexed to polyethylenimine and administrated to neonatal ducklings via the i.v route were able to drastically inhibit hepadnaviral replication [18].

The pregenomic RNA and hepadnaviral encapsidation signal ε play an important role in initiation of hepadnaviral reverse transcription (RT), and they appear as interesting targets for antiviral therapy [53,54]. To explore the capacity of anti-ε PNAs to inhibit viral replication, we first investigated their antiviral activity in vitro, in a cell-free system for enzymatically active DHBV RT expression. Our results indicated that PNA that targeted the bulge and upper stem of epsilon exhibited a potent and sequence-specific inhibitory effect on DHBV reverse transcriptase [8].

Next, to investigate the in vivo antiviral effect of PNAs, we coupled the anti-ε PNA to a cationic CPP (D-Arg)_8_ to increase its cellular uptake. Indeed, the main difficulty in the use of PNAs as antivirals is their poor transport across the cell membrane and their limited intracellular biodistribution. To optimize the in vivo delivery of PNA-CPP conjugates and their bioavailability, we administrated PNA-CPP coupled to FITC or PNA-FITC in vivo to DHBV-free neonatal ducklings. The hepatocyte-associated fluorescence was analyzed by fluorescence microscopy 48 hours later. The results showed little or no hepatocyte-associated fluorescence after i.v injection of FITC-PNA not conjugated to (D-Arg)_8_, whereas the injection of FITC-CPP-PNA resulted in a high level of hepatocyte-associated fluorescence. This finding indicates that (D-Arg)_8_ CPP is able to efficiently deliver anti-ε PNA to the liver following intravenous injection ([10] and unpublished observations). Moreover, the i.v injection of a FITC-PNA(D-Arg)_8_ to ducklings was more effective than the intraperitoneal (i.p.) route as only following i.v administration was the FITC-PNA detected in the hepatocytes [10]. This differs from the mouse model in which i.p. injection resulted in better delivery of PNAs. In addition, our findings demonstrated for the first time that intravenous administration of CPP-PNA conjugates to ducklings, i.e. larger animals than mice, resulted in an effective delivery of these conjugates to hepatocytes that was associated with an antiviral effect [10].

We next asked whether such anti-ε PNA conjugated to (D-Arg)_8_ inhibits hepadnaviral replication in vivo in the DHBV infection model [10]. Thus, DHBV-infected ducklings were treated with this (D-Arg)_8_-PNA conjugate (1 μg/g body weight/day) for six days. At the end of this treatment, the analysis of serum and liver DNA revealed a marked decrease in viremia and liver DHBV DNA, i.e. a decrease of about 50% in treated animals compared with the untreated controls (Table 1).

Surprisingly, 2 nt mismatched PNA coupled to the same (DArg)_8_ CPP and used as control also inhibited viral replication. Therefore, an antiviral activity of (D-Arg)_8_ was suspected. Indeed, the i.v administration of (DArg)_8_ alone to DHBV-infected ducklings resulted in a decrease in viremia, confirming the ability of this CPP to inhibit hepadnaviral replication (as detailed above). Altogether, these findings indicate that anti-DHBV activity exhibited by (DArg)_8_ CPP alone may explain the limited sequence specificity of this CPP-PNA conjugate. No loss of weight was observed in all treated animals compared with the untreated control animals during the six days of treatment (data not shown), showing the absence of in vivo toxicity of (DArg)_8_-PNA conjugates and of (DArg)_8_ alone.

To better understand the role of CPPs used as a vehicle in delivery of PNAs, we examined the antiviral activity of the same anti-ε PNA but conjugated to another cationic CPP, (D-Lys)_4_ in vitro, in PDH. Moreover, the antiviral activity of this (D-Lys)_4_ CPP alone was analyzed. Thus, treatment of DHBV-infected PDH by (D-Lys)_4_-PNA conjugates led to a marked inhibition of DHBV release in cell culture supernatants and to a decrease in intracellular viral replication.

Interestingly, this inhibition was specific, since a 2-nucleotides mismatched PNA conjugated to the same (D-Lys)_4_ CPP, displayed no pronounced inhibitory activity on DHBV secretion and on intracellular DHBV DNA. Importantly, the treatment of the same cells by (D-Lys)_4_ CPP alone did not affect viral replication [10]. Remarkably, neither (D-Lys)_4_ and its corresponding bonding CPP-PNA conjugates displayed toxicity in PDH cells, as assessed by Neutral Red test [10].

Collectively, these findings demonstrate that the antiviral activity of some cationic CPPs such as (DArg)_8_, used as vehicles to improve cellular uptake of their bioactive PNA cargos, may reduce the sequence-specificity of CPP-PNA conjugates. In contrast, in the absence of the inhibitory effect exhibited by these cationic CPPs, the CPP-PNA conjugates are able to specifically inhibit hepadnaviral replication [10].

Other preclinical studies demonstrated the benefit of CPP or PTD peptides used as vehicles. Indeed, PTD-p53 conjugates effectively suppressed production of HBV messenger RNAs (mRNAs), as well as HBsAg, HBeAg, and HBcAg, both in vitro and in vivo [55]. Also, CTP peptides improved Tapasin uptake and enhanced cytotoxic T lymphocyte activity [56]. Interestingly, the single-chain antibody targeting the hepatitis B core protein and coupled to the CPP was internalized into HepG2.2.15 cells and inhibited HBV replication in vitro [25].

## 5. Sugar Modified CPP-PNA Uptake and Their Anti-HBV Activity in HepaRG Cells

The asialoglycoproteins receptors (ASGP-R) were the first mammalian lectins described [57] and are found on the hepatocellular membranes. Thus, ASGP-R are known to facilitate the uptake into the hepatocytes through receptor-mediated endocytosis of a variety of ligands such as glycoproteins having a galactose-terminal carbohydrate. Indeed, the d-galactose-(poly)Lys- bovine serum albumin (BSA) complex improved the intracellular expression of the IX human factor gene in HepG2 cells [58]. Moreover, PNAs uptake by hepatocytes was enhanced following their incubation with an asialofetuin–oligonucleotide complex (AF/ODN) [59]. In addition, the sugar-dependent nuclear import of neoglycoproteins was shown to increase the expression of transferred genes [60].

In another study, PNAs hybridized to the AF/ODN conjugates were efficiently internalized into murine primary hepatocytes and in HepG2 cells through receptor-mediated endocytosis. Indeed, after a 4-h incubation, PNAs were mainly localized in the nuclei of the cells. In addition, more than 70% inhibition of telomerase activity was detected when anti-human telomerase PNA was carried to HepG2 cells using AF/ODN. An in vivo study showed that the AF/ODN-PNAs conjugates led to an effective delivery of PNAs to the liver after intravenous injection into mice [61].

We evaluated the anti-HBV activity of sugar-modified CPP-PNA conjugates in the HepaRG human hepatoma cell line. We chose HepaRG cells for this study since these cells when differentiated share different common features with human hepatocytes, including the expression of ASGP-R on their surface. Differentiated HepaRG cells can be effectively infected with HBV, reproducing a full viral replication cycle and cccDNA formation. Moreover, HepaRG are considered as a reference in vitro cell culture model for anti-HBV drug evaluation [62,63].

**Figure 1:**
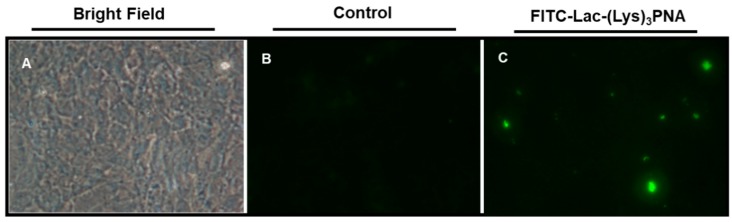
Uptake of FITC-Lac-PNA in HepaRG cells. HepaRG cells were plated and incubated at 37 °C, 5% of CO_2_ with FITC-Lac-(Lys)3-PNA conjugates for 24 h. At the end of treatment, cells were observed by fluorescence microscopy (**A**) Bright field; (**B**) Control cells; (**C**) FITC-Lac-(Lys)3-PNA. FITC = fluorescein isothiocyante; Lac = lactose; PNA = peptide nucleic acids; Lys = lysine.

Therefore, we investigated in differentiated HepaRG cells whether a PNA that targets an HBV envelope protein coupled to a Lactose and Lysine, (Lac-(Lys)_3_-PNA), could HBsAg release. We first investigated the cellular uptake of Lac-(Lys)_3_-PNA coupled to FITC that was incubated with uninfected HepaRG cells. Our preliminary results showed uptake of fluorescein-labeled PNA indicating that Lac-CPP-PNA conjugates can enter into the HepaRG cells (Figure 1).

**Figure 2:**
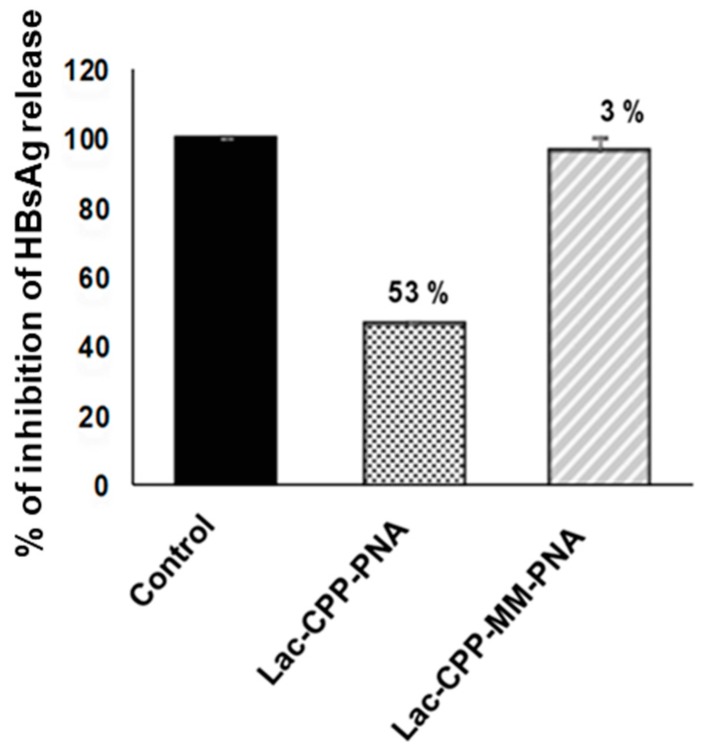
Antiviral effect of Lac(Lys)3-PNA conjugates in vitro, in HBV-infected cells. HepaRG, human hepatocyte like cells, were plated and infected with HBV followed by treatment with Lac(Lys)3-PNA (Lac-CPP-PNA) or with its corresponding mismatch control (Lac-CPP-MMPNA). The cell culture supernatants were harvested daily and Hepatitis B surface antigen (HBsAg) was quantified. Untreated HBV-infected control cells were considered as 100% and percentages of inhibition are indicated above the bars. The error bars display the standard deviation of duplicates.

Next, the antiviral effect of this Lac-(Lys)_3_-PNA conjugate was evaluated in HBV-infected HepaRG cells. As illustrated in Figure 2, a marked decrease in the release of HBsAg into cell culture supernatants was observed, which was estimated at 53% inhibition as compared with untreated controls. Moreover, the inhibition was specific since a 2-nt mismatched PNA conjugated to the same vehicle (Lac-(Lys)_3_-mismatch peptide nucleic acid (MM PNA)) showed a far less marked (3%) inhibitory effect on the release of HBsAg.

Our preliminary results suggest an uptake of Lac-(Lys)_3_-PNA into hepatocytes via interaction of the Lactose moiety with ASGP-R, probably through receptor-mediated endocytosis. Altogether, these data demonstrate that treatment of HBV-infected HepaRG cells by anti-S PNA coupled to Lactose increases its uptake and decreases HBsAg release, although internalization and inhibitory potential of this novel conjugate need to be further investigated.

## 6. Antiviral Activity of CPP-Based Approaches against Other Viruses

In addition to hepadnaviruses, the antiviral activity of CPPs, CPP-ODN, or CPP-protein conjugates targeting other viruses was investigated. Indeed, a monoclonal antibody targeting the HIV-1 capsid protein (p24), chemically conjugated to κFGF-MTS CPP, entered into HIV-1-infected cells and inhibited viral replication up to 73 and 49% in T-lymphocyte and in primary peripheral blood mononuclear cells (PBMCs), respectively [64].

In another study, a modified CPP, derived from the HIV-1 TAT protein, exhibited a potent anti-HIV-1 activity in vitro. Importantly, treatment of HIV-1 infected cells with this CPP showed a direct relationship between its cationic charge and antiviral potency [65].

Interestingly, a chimeric peptide (FP-PTD) was able to deliver specific siRNA (FP-PTD-siRNA) into HIV-1-infected cells and to efficiently inhibit viral replication [66].

In another report, Yoo et al., showed that a CPP-PNA conjugate targeting the top of the 3′-untranslated transcribed region of massager RNA (3′-UTR) loop of the Japanese encephalitis virus (JEV) structure was effective in blocking viral proliferation [67].

Coupling of artificial zinc-finger proteins (AZPs) to CPPs led to CPP-AZP conjugates, which appeared as pertinent antiviral agents against Human papillomavirus (HPV). Indeed, several studies demonstrated that greater internalization of CPP-AZP conjugates into mammalian cells resulted in more potent inhibition of viral replication [68,69]. Thus, an increasing amount of data indicate that CPP-based approaches are of particular interest as antiviral strategies.

## 7. Conclusions

Taken together, different studies demonstrated an important role of CPPs or modified-CPPs in the intracellular delivery of PNAs that target the hepadnaviral replication pathway. Indeed, in the DHBV infection model, PNA that targets the viral encapsidation ε signal and is coupled to CPPs decreased viral replication in vitro in PDHs and in vivo in ducklings. Interestingly, some CPPs, (D-Arg)_8_ and Decanoyl-(D-Arg)_8_, alone displayed a potent antiviral effect, altering the late stages of DHBV and HBV morphogenesis. In addition, lipid and sugar domain-conjugated CPPs delivered their PNA cargos into hepatocytes and displayed an increased antiviral activity. Importantly, an anti-HBV PNA coupled to Lactose exhibited a high affinity to the asialoglycoprotein receptor on hepatocellular membranes and was shown to enter into the human hepatoma HepaRG cells and inhibit HBsAg release.

Thus, these different preclinical studies provide the evidence that CPPs, modified-CPP, and CPP-PNAs conjugates represent pertinent molecular tools for future development of novel therapeutic approaches to fight chronic hepatitis B infection. Further studies are warranted to explore the antiviral potency of these compounds, targeting different steps of hepadnaviral replication, which could be used in combination with immune therapies able to break immune tolerance in HBV-carrier patients and to enhance viral infection clearance.

Moreover, CPPs and modified CPPs appear of promise for development of novel antiviral strategies against different RNA and DNA viruses.

## Figures and Tables

**Table 1:** Evaluation in vitro and in vivo of cell penetrating peptides (CPP)-based approaches targeting hepadnaviral replication. PDH = primary duck hepatocytes; DHBV = duck hepatitis B virus.

CPPs	Cell or Animal Models	Target	% D’inhibition	Toxicity
(DArg)_8_	PDHs	DHBV	44–60% (2 μM)	[10,23]
	Pekin ducklings	DHBV	42% (1 μg/g/bw/day)	– [10]
Decanoyl(DArg)_8_	PDHs	DHBV	86–90% (2 μM)	– [23]
	LMH-D2	DHBV	88–90% (2 μM)	– [23]
	HepG2.2.15	HBV	50% (4 μM)	– [23]
(DArg)_8_-PNA	PDHs	DHBV Epsilon (ε)	47–59% (2 μM)	– [10]
	Pekin ducklings	DHBV Epsilon (ε)	49–59% (1 μg/g/bw/day)	– [10]

Inhibition of DHBV and hepatitis B virus (HBV) replication in cells and in animal models by CPPs, modified CPPs, or CPP-PNAs. In vitro DHBV-infected PDH, stably DHBV-transfected LMH-D2, or HBV-transfected HepG2.2.15 cells were treated with 2 μM of (DArg)_8_, Decanoyl (DArg)_8_, or (DArg)_8_-PNA for 5 to 6 days. The impact of treatment on viral replication was monitored by analysis of viral DNA in cell culture supernatants, collected daily, and of intracellular viral DNA at the end of treatment. In vivo DHBV-infected Pekin ducklings were treated with 1 μg/g/bw/day of (DArg)_8_ or (DArg)_8_-PNA for 6 days, and then serum and intrahepatic DHBV DNA was analyzed. Viral DNA was detected by hybridization with a DHBV or HBV32P-labeled probe. Viral DNA was quantified by PhosphorImager scanning and was expressed as percentage of inhibition, considering untreated controls as 100%. Percentages of inhibition from at least two independent experiments are indicated. Cellular toxicity was appreciated by (3-(4,5-dimethylthiazol-2-yl)-2,5-diphenyltetrazolium bromide (MTT) assay after daily incubation with different compounds, and in vivo toxicity was measured by animal weight monitoring [10,23].
